# The influence of docetaxel schedule on treatment tolerability and efficacy in patients with metastatic breast cancer: a systematic review and meta-analysis of randomized controlled trials

**DOI:** 10.1186/s12885-022-09196-x

**Published:** 2022-01-25

**Authors:** Maarten van Eijk, Marit A. C. Vermunt, Erik van Werkhoven, Erica A. Wilthagen, Alwin D. R. Huitema, Jos H. Beijnen

**Affiliations:** 1 Department of Pharmacy & Pharmacology, The Netherlands Cancer Institute - Antoni van Leeuwenhoek, Plesmanlaan 121, 1066 CX Amsterdam, The Netherlands; 2 Department of Biometrics, The Netherlands Cancer Institute - Antoni van Leeuwenhoek, Plesmanlaan 121, 1066 CX Amsterdam, The Netherlands; 3 Scientific Information Service, The Netherlands Cancer Institute - Antoni van Leeuwenhoek, Plesmanlaan 121, 1066 CX Amsterdam, The Netherlands; 4 Department of Clinical Pharmacy, University Medical Center Utrecht, Utrecht University, Heidelberglaan 100, 3584 CX Utrecht, the Netherlands; 5 Department of Pharmacology, Princess Máxima Center for Pediatric Oncology, Heidelberglaan 25, 3584 CS Utrecht, the Netherlands; 6 Division of Pharmacoepidemiology & Clinical Pharmacology, Science Faculty, Utrecht Institute for Pharmaceutical Sciences (UIPS), Utrecht University, Heidelberglaan 8, 3584 CS Utrecht, The Netherlands

**Keywords:** Docetaxel, Schedule, Metastatic breast cancer, Neutropenia

## Abstract

**Background:**

Administration of single-agent docetaxel in a weekly schedule may offer similar efficacy, with a more favorable toxicity profile, compared to a three-weekly schedule in patients with metastatic breast cancer.

**Methods:**

The original search of Medline, Embase, and Scopus was performed in September 2018 and references were updated with additional searches up to January 2021. Two reviewers independently screened the identified literature based on a predefined set of criteria.

Randomized controlled trials investigating the use of weekly versus three-weekly docetaxel in metastatic breast cancer patients were included.

**Results:**

Four randomized controlled trials (*N* = 459 patients) were included in the final analyses. No significant differences were found in terms of objective response rate (risk ratio (RR) 0.75, 95% confidence interval (CI): 0.54 – 1.05), progression-free survival (hazard ratio (HR) 0.95, 95% CI: 0.71 – 1.26) or overall survival (HR 0.95, 95% CI: 0.70 – 1.29) between weekly and three-weekly docetaxel, respectively. Weekly docetaxel was associated with a significantly lower risk of grade 3/4 neutropenia (RR 0.16, 95% CI: 0.10 – 0.27), febrile neutropenia (RR 0.21, 95% CI: 0.08 – 0.55), and neuropathy (RR 0.29, 95% CI: 0.11 – 0.78). Although the risk of epiphora (≥ grade 3/leading to treatment withdrawal, RR 3.62, 95% CI: 1.07–12.22) and onycholysis (≥ grade 2/leading to treatment withdrawal, RR 3.90, 95% CI: 1.34 – 11.32) was increased.

**Conclusions:**

Weekly docetaxel is associated with a lower risk of neutropenia, febrile neutropenia and neuropathy than the three-weekly docetaxel schedule in metastatic breast cancer patients. However, the risk of onycholysis, epiphora, and treatment discontinuation seems increased with weekly administration. No significant differences in efficacy outcomes were found. Weekly docetaxel might be an alternative for patients at risk for developing neutropenia.

**Supplementary Information:**

The online version contains supplementary material available at 10.1186/s12885-022-09196-x.

## Background

Breast cancer is, and has remained, the most commonly diagnosed cancer in women worldwide [1]. Most patients with metastatic breast cancer (mBC) have recurrent metastatic disease after primary treatment for earlier-stage breast cancer but a small fraction of patients present with de novo metastatic disease [2]. MBC is currently considered incurable. For this reason, therapeutic goals in mBC are mainly prolongation of survival, maintenance of the quality of life and palliation of symptoms.

Docetaxel is applied in mBC, either in combination with capecitabine after failure of first-line anthracycline-based treatment, in combination with HER2-targeted therapy in HER2-positive mBC, or as monotherapy in second or later palliative lines [3–5].

Docetaxel is generally administered as an 1-h infusion of 75–100 mg/m^2^ in the USA and Europe, while patients in Asia receive a lower standard dose of 60 mg/m^2^ every three-weeks [5, 6]. For this three-weekly schedule, myelosuppression with potentially life-threatening febrile neutropenia, is observed as the main dose limiting toxicity, with incidences increasing with the dose [7]. In an attempt to reduce this toxicity, a weekly docetaxel schedule has been investigated in several phase I and II trials. In most phase II studies, weekly docetaxel was given in doses of 30–40 mg/m^2^/week for 6 weeks in an 8-week cycle or for three-weeks in a four-week cycle. These studies were evaluated in multiple reviews to provide an indirect comparison of weekly and three-weekly docetaxel in mBC patients [8–13]. Although less myelosuppression was observed with the weekly schedule, chronic toxicities such as fatigue, asthenia, nail toxicity, fluid retention and lacrimation seemed more profound with weekly docetaxel. Objective response rates ranging from 13% to 86.7% in these phase II studies indicated promising efficacy of weekly docetaxel in mBC [8–13].

In view of the mild acute toxicity profile, most authors considered weekly docetaxel as a reasonable alternative for the three-weekly regimen, especially for elderly or unfit patients [8–12]. Other authors preferred the three-weekly regimen and viewed weekly docetaxel as a back-up schedule for individual vulnerable patients only, as the chronic toxicities and the burden of more frequent hospital visits might negatively affect the quality of life for the general mBC population [13].

The objective of this systematic review and meta-analysis was to systematically identify and analyze efficacy and toxicity data from randomized clinical trials (RCTs) comparing weekly and three-weekly single-agent docetaxel schedules in patients with mBC.

## Methods

### Search and eligibility criteria

The original searches were performed in the electronic databases Medline (Ovid), Embase (Ovid), and Scopus in September 2018. Subsequent updated searches in these databases were conducted in September 2019 and January 2021. The search strategy included (“breast cancer” AND “docetaxel” AND “survival rate/response rate”) and included combinations of free-text keywords, equivalent words in title/abstracts, and standardized keywords (MeSH and Emtree). The applied search strategy is included in the supplementary material (Additional file 1). Duplicate articles were removed according to the method of Bramer et al. [14]. The literature screening process was performed independently by two authors (ME and MV). RCTs that investigated efficacy and/or toxicity outcomes in patients with mBC that were treated with weekly versus three-weekly single-agent docetaxel were considered eligible for inclusion. Reports not describing a randomized prospective clinical trial were excluded, as were studies not directly comparing weekly and three-weekly single-agent docetaxel schedules, studies not conducted in patients with mBC, studies investigating adjuvant or neoadjuvant docetaxel treatment, studies not reporting either toxicity or efficacy outcomes, preclinical studies, review articles, letters, case reports, and conference abstracts with insufficient details or for which a more recently published journal article was available. References were initially screened based on title and abstract. If agreement on eligibility was achieved between two reviewers, a full-text review of the publication was performed. Full-text review was again performed blinded and independently by the same two authors. In addition, references of the included RCT’s and previous systematic reviews were hand-searched for potentially eligible publications that had not been identified in the initial search.

### Data extraction and statistical analysis

Data extraction was performed by one author (ME), and verified by another (MV). Objective response was treated as a dichotomous outcome and the risk ratio (RR) was calculated in an intention-to-treat approach. Similarly, toxicities were treated as dichotomous outcomes and analyzed using RR. However, the safety population for the calculation of the RR for different toxicities was composed of patients who actually received the allocated intervention. Time-to-event outcomes were analyzed using hazard ratio’s (HR). If not directly available from the published journal article, the HR and corresponding variance were calculated using the methods described by Tierney and colleagues [15]. Dichotomous outcomes were analyzed using a random-effects meta-analysis of the RR using the Mantel–Haenszel method [16]. For time-to-event outcomes the pooled HR was estimated with a random-effects model weighing the inverse of the variance [17]. In both cases between-study variance was represented by the variance of the distribution of the observed study effects (τ^2^) which was estimated with a DerSimonian-Laird estimator. Cochran’s Q test was used to test for heterogeneity. Due to the low number of included studies, the p-value for significance of this test was set to 0.10 [17].The I-square statistic (I^2^) was used as a measure for statistical heterogeneity for which a value greater than 50% is considered high heterogeneity [17]. The conventional level of 0.05 (two-sided) was used for all other tests. All analyses were conducted in R (version 4.0.3), using the meta package [18, 19].

### Risk of bias

We assessed risk of bias in the included RCTs using the Cochrane Collaboration Risk of Bias 2 tool [20]. Two authors (ME and MV) independently completed the risk of bias assessments for the following domains: 1) Randomization process; 2) Deviations from intended interventions; 3) Missing outcome data; 4) Measurement of the outcome; 5) Selection of the reported result. The risk of bias per domain was classified as either ‘low’, ‘some concerns’, or ‘high’. Final decision on the risk of bias was made after consensus between both assessors.

## Results

### Included studies and characteristics

**Fig. 1:**
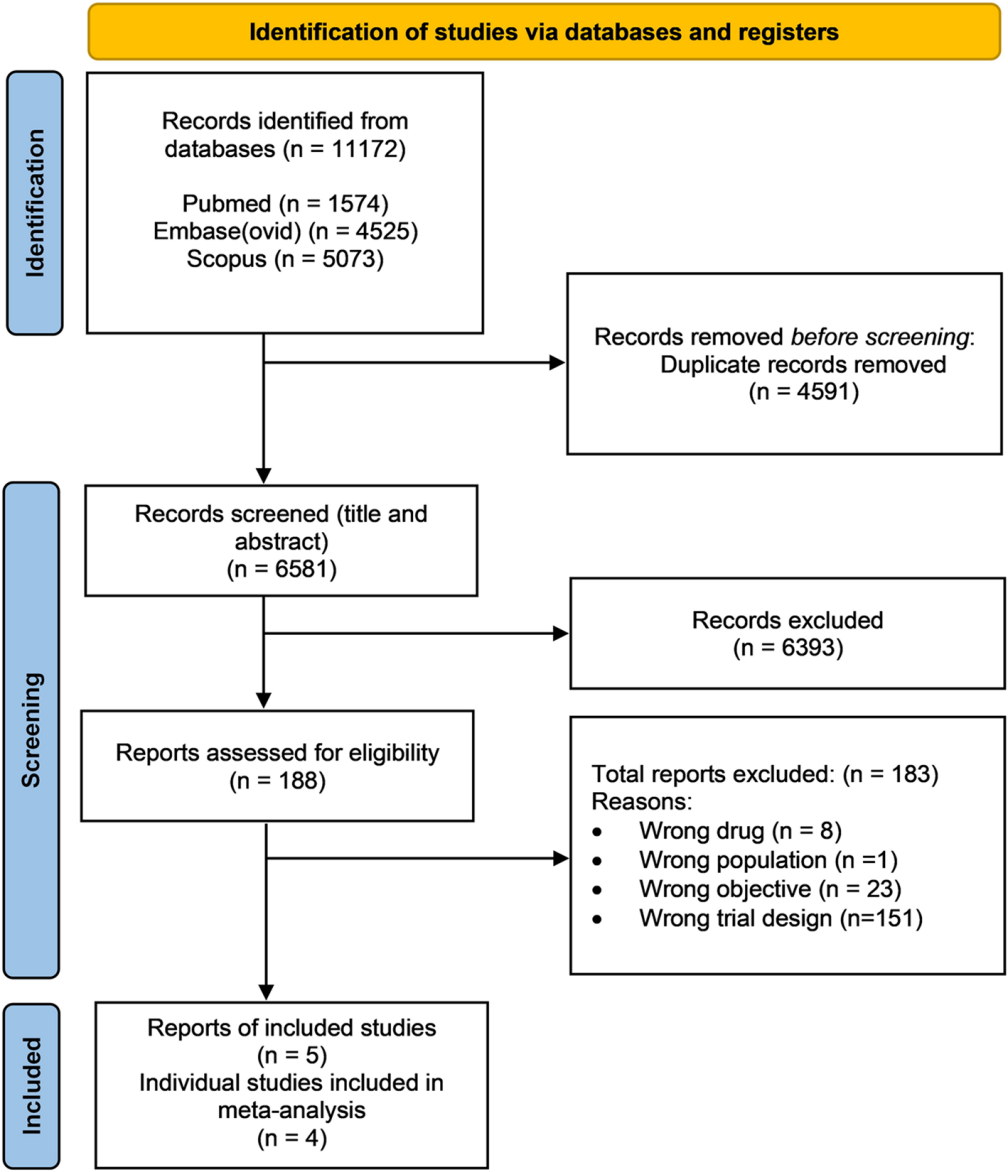
PRISMA flow diagram of the systematic literature search

The Preferred Reporting Items for Systematic Reviews and Meta-Analyses (PRISMA) flow diagram of the literature search is shown in Fig. 1 [21]. After the exclusion of duplicates, the original and the updated searches identified 6581 publications to be screened for eligibility based on title and abstract. From this selection, 188 publications were included for full text review. Ultimately, 4 RCTs were included for meta-analysis [22–25]. The characteristics of these studies are described in Table 1. In total, these studies included 459 evaluable patients of which 227 had received weekly docetaxel and 232 had received docetaxel every three-weeks.

**Table 1:** Characteristics of included randomized controlled trials

**Characteristics**	**Tabernero et al.** [22]	**Rivera et al.** [23]	**Stemmler et al.** [24]	**Schröder et al.** [25]
**Year of publication**	2004	2008	2010	2011
**Population**	mBC	mBC	mBC	mBC
**Phase**	Phase II	Phase III	Phase III	Phase III
**Total patients Randomized**	83	125	102	161
**Total patients Evaluable**	82, 83 ^c^	118	102	156, 143 ^c^
Weekly arm (QW)	41	59	48	79, 81 ^c^
Three-weekly arm (Q3W)	41, 42 ^c^	59	54	77, 78 ^c^
**Breast cancer subtype**				
Oestrogen receptor positive	QW: 56.1% Q3W: 52.4%	QW: 55.6% Q3W: 48.4%	QW: 75.0% Q3W: 64.8%	QW: unknown Q3W: unknown
Her-2 positive	QW: unknown Q3W: unknown	QW: 4.7% Q3W: 12.9%	QW: 12.5% Q3W: 11.1%	QW: unknown Q3W: unknown
Triple negative	QW: unknown Q3W: unknown	QW: unknown Q3W: unknown	QW: unknown Q3W: unknown	QW: unknown Q3W: unknown
**Prior anthracyclines**	QW: 82.9% Q3W: 78.6%	QW: 61.9% Q3W: 66.1%	QW: 20.8% Q3W: 29.6%	QW: 95.1% Q3W: 93.6%
**Age** (median (range))	QW: 56 (25–75) Q3W: 55 (33–72)	QW: 54 (32–86) Q3W: 56 (36–82)	QW: 73 (58–84) Q3W: 71 (60–82)	QW: 56 (29–74) Q3W: 53 (30–79)
**Performance status**	QW: 61% PS0, 34% PS1, 5% PS2 ^d^ Q3W: 57% PS0, 38% PS1, 2% PS2 ^d^	QW: NR Q3W: NR	QW: median 80%, range 60–100% ^e^ Q3W: median 80%, range 60–100%^e^	QW: 35% PS0, 47% PS1, 19% PS2 ^d^ Q3W: 28% PS0, 51% PS1, 21% PS2 ^d^
**Primary endpoint**	Toxicity	Response and toxicity	Hematological toxicity	Toxicity
**Accrual period**	23 months November 1999 – October 2001	44 months January 2001 – September 2004	85 months July 2001 – August 2008	62 months February 2001 – April 2006
**Median follow-up**	10 mo (95% CI 0.5–24 mo) (QW) 10.2 mo(95% CI 0.3–26.7 mo)(Q3W)	15.1 mo (range 0.5—51.6 months)	14.4 months ^b^ (range 1.2—77.7 months)	8.9 months (range, not reported)
**Docetaxel treatment**				
QW	40 mg/m^2^ Weekly for 6 weeks, 2 weeks rest	35–40 mg/m^2^ Weekly for 3 weeks, 1 week rest	30 mg/m^2^ Weekly for 3 weeks, 1 week rest	36 mg/m^2^ Weekly for 6 weeks, 2 weeks rest
Q3W	100 mg/m^2^ Every 3 weeks	75–100 mg/m^2^ Every 3 weeks	75 mg/m^2^ Every 3 weeks	100 mg/m^2^ Every 3 weeks
**Dexamethasone dose** ^f^	QW: 24 mg Q3W: 48 mg	QW: 12 mg Q3W: 48 mg	QW: NR Q3W: NR	QW: 16 mg Q3W: 32 mg

*mBC* Metastatic breast cancer, *NR* Not reported, *PS *ECOG Performance Status, *QW* Once weekly arm, *Q3W* Three-weekly arm

^a^Percentage of patients that were treated with prior anthracycline containing chemotherapy, either in (neo)adjuvant or metastatic setting

^b^Defined as median observation time

^c^Number of patients evaluable for toxicity/efficacy respectively

^d^Eastern Cooperative Oncology Group performance status

^e^Karnofsky Performance status

^f^Total dose of dexamethasone administered with every single docetaxel administration

In the first study, published by Tabernero and colleagues in 2004, 83 patients with mBC were randomized to receive weekly or three-weekly docetaxel [22]. One previous line of palliative chemotherapy was allowed, including paclitaxel, except for patients with recurrent disease within 1 year after completion of adjuvant treatment. For 85.4% of the patients in the weekly arm and 78.6% in the three-weekly arm, docetaxel was their first palliative line of chemotherapy. However, 82.9% and 78.6% had received prior anthracyclines with or without a taxane in the weekly and three-weekly arm, respectively. In the weekly arm, patients received a weekly docetaxel dose 40 mg/m^2^ continuously for 6 weeks, followed by 2 weeks of interruption. The patients in the three-weekly arm were treated with a docetaxel dose of 100 mg/m^2^ every 21 days. Oral dexamethasone was given at a dose of 48 mg every three-weeks in the three-weekly arm, while patients in the weekly arm received a total dexamethasone dose of 24 mg at each docetaxel infusion.

The second study, published by Riviera and colleagues in 2008, was conducted in 118 mBC patients who were pretreated with a maximum of 1 prior palliative chemotherapy regimen, in which docetaxel was not allowed [23]. Docetaxel was the first palliative line of chemotherapy for 71% of the patients in the weekly arm and 69% in the three-weekly arm. Of all patients, 38.1% in the weekly and 33.9% in the three-weekly arm had never received prior anthracycline-based treatment. The patients randomized to the three-weekly arm started with a docetaxel dose of 75 mg/m^2^ which was increased at cycle 2 to 100 mg/m^2^, unless grade 4 neutropenia lasting > 7 days or associated with fever or infection was observed. The patients in the weekly arm were treated with a starting dose of 35 mg/m^2^ for three-weeks followed by one week rest, which was increased to 40 mg/m^2^ unless neutropenia occurred as described for the three-weekly arm. Patients in the three-weekly arm were premedicated with oral dexamethasone in a total dose of 48 mg every three-weeks. Patients in the weekly arm received a total dexamethasone dose of 12 mg with each docetaxel administration.

The third study, published by Stemmler and colleagues in 2010, compared both schedules in 102 mBC patients [24]. All patients were required to have a Karnofsky Performance status of 60–80% or an age ≥ 60 years. No prior palliative chemotherapy was allowed and only 20.8% and 29.6% of the patients had received prior anthracycline-containing adjuvant chemotherapy in the weekly and three-weekly arm, respectively. Patients in the three-weekly arm were treated with docetaxel dose of 75 mg/m^2^, while patients in the weekly arm received docetaxel 30 mg/m2 on day 1, 8 and 15, followed by 1 week rest. Corticosteroid premedication was given according to local standards and the dose was not further specified.

In the fourth study, published by Schröder and colleagues in 2011, 162 mBC patients were randomized to either weekly or three-weekly docetaxel [25]. One prior line of non-taxane chemotherapy was allowed. Of all patients, 65.4% and 69.2% had received chemotherapy in metastatic disease setting in the weekly and three-weekly arm, respectively. Almost all patients were pretreated with anthracycline containing chemotherapy. Around 20% of the patients in the trial had a relatively low baseline WHO performance status of 2. Treatment dose in the weekly arm was 36 mg/m^2^ continuously for 6 weeks, followed by 2 weeks of rest. Patients in the three-weekly arm received a docetaxel dose of 100 mg/m^2^. In the weekly docetaxel arm, patients received twice-daily dexamethasone premedication on the day of docetaxel administration in a total dose of 16 mg, as intravenous gift at 30 min before the docetaxel administration and as one oral gift on the evening of the infusion day. For the three-weekly arm, dexamethasone was given for 2 days in a daily dose of 16 mg, starting 1 day before infusion.

### Hematological toxicity

The pooled risk ratios of the most important hematological and non-hematological toxicities, along with their 95% confidence interval (CI), are shown in Table 2. In our random-effects meta-analysis, the weekly schedule was associated with a substantially lower relative risk of ≥ grade 3 neutropenia and febrile neutropenia compared to the three-weekly regimen (RR 0.16, 95% CI: 0.10—0.27, *p* < 0.001; RR 0.21, 95% CI: 0.08 – 0.55, *p* < 0.01).

**Table 2:** Results of meta-analyses of toxicities reported in randomized controlled trials with weekly versus three-weekly docetaxel

Toxicity	No. studies	Ref	Weekly Totals n/N	Three-weekly Totals n/N	Pooled RR	95% CI	p	I^2^ (%)	P_q_	τ^2^
**Grade 3/4**										
Neutropenia	3	[22, 23, 25]	14/179	87/177	0.16	0.10 – 0.27	< 0.001	0	0.67	0
Febrile Neutropenia	3	[22, 23, 25]	5/179	27/177	0.21	0.08 – 0.55	< 0.01	0	0.48	0
Neuropathy	4	[22–25]	5/227	20/231	0.29	0.11 – 0.78	0.01	0	0.66	0
Infections	3	[22, 24, 25]	10/168	9/172	1.11	0.45 – 2.73	0.82	0	0.62	0
Fatigue/Asthenia	3	[22, 23, 25]	23/179	28/177	0.81	0.48–1.37	0.44	1	0.36	0.003
Fluid retention/edema/effusions	4	[22–25]	13/227	8/231	1.49	0.44 – 5.02	0.52	31	0.23	0.472
Skin toxicity	3	[22, 24, 25]	19/168	9/172	2.04	0.75 – 5.56	0.16	34	0.22	0.269
Nausea ^a^	4	[22–25]	11/227	15/231	0.74	0.31–1.78	0.50	12	0.33	0.094
Vomiting ^a^	4	[22–25]	12/227	14/231	0.85	0.30–2.37	0.75	32	0.22	0.358
Diarrhea	3	[22, 24, 25]	8/168	17/172	0.50	0.22–1.14	0.10	0	0.67	0
** ≥ Grade 3 or leading to treatment withdrawal**										
Epiphora/lacrimation	2	[22, 23]	13/100	3/100	3.62	1.07–12.22	0.04	0	0.47	0
** ≥ Grade 2 or leading to treatment withdrawal**										
Onycholysis ^b,c^	2	[22, 25]	16/120	4/118	3.90	1.34 – 11.32	0.01	0	0.79	0
**Other**										
Patient withdrawals due to toxicity	2	[22, 25]	43/120	27/118	1.52	1.00 – 2.32	0.05	11	0.29	0.011

*n* number of patients with an event in the treatment arm, *N* Total number of patients in the treatment arm, *RR* risk ratio, *CI* confidence interval, *P*_*q*_ *p*-value for heterogeneity, *I*^*2*^ measure of statistical heterogeneity, *τ*^*2*^ between-study variance

^a^Events from studies where combined outcome was reported cases were counted in both separate analyzed toxicities

^b^Grade cut off based on largest group available for analysis

^c^Ref. [23] not included in analysis since no grade 2 onycholysis reported

### Non-hematological toxicity and treatment withdrawals

As shown in Table 2, weekly docetaxel was associated with a significantly lower risk of neuropathy (RR 0.29, 95% CI: 0.11 – 0.78, *p* = 0.01). The weekly schedule was, on the other hand, associated with a higher risk of epiphora or lacrimation (≥ grade 3 or leading to treatment withdrawal, RR 3.62 95% CI: 1.07 – 12.22, *p* = 0.04) and onycholysis (≥ grade 2 or leading to treatment withdrawal, RR 3.90 95% CI: 1.34 – 11.32, *p* = 0.01). The rate of treatment discontinuation was reported in two trials [22, 25]. Meta-analysis of this outcome indicated a borderline significant association between increased risk of treatment discontinuation and the weekly schedule (RR 1.52, 95% CI: 1.00 – 2.32, *p* = 0.05).

### Efficacy

**Fig. 2:**
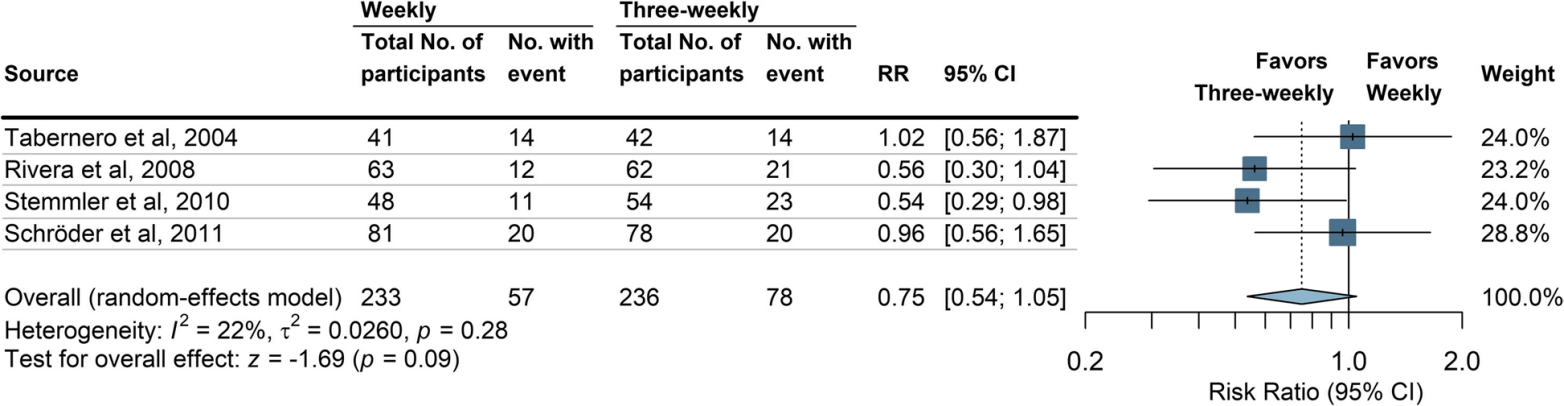
Random-effects meta-analysis of objective response rate comparing weekly and three-weekly docetaxel schedules; squares demonstrate the point estimate of the risk ratio in each study; lines represent the 95% CI; the size of each square is proportional to its weight in meta-analysis; the diamond represents the pooled estimate of the risk ratio after meta-analysis; values < 1 indicate a lower ‘risk’ of response in the weekly arm as opposed to the three-weekly arm while values > 1 indicate a higher ‘risk’ of response in the weekly arm; I^2^ measure of statistical heterogeneity with the corresponding p-value as test for heterogeneity across studies; τ^2^ variance of the distribution of the observed study effects

**Fig. 3:**
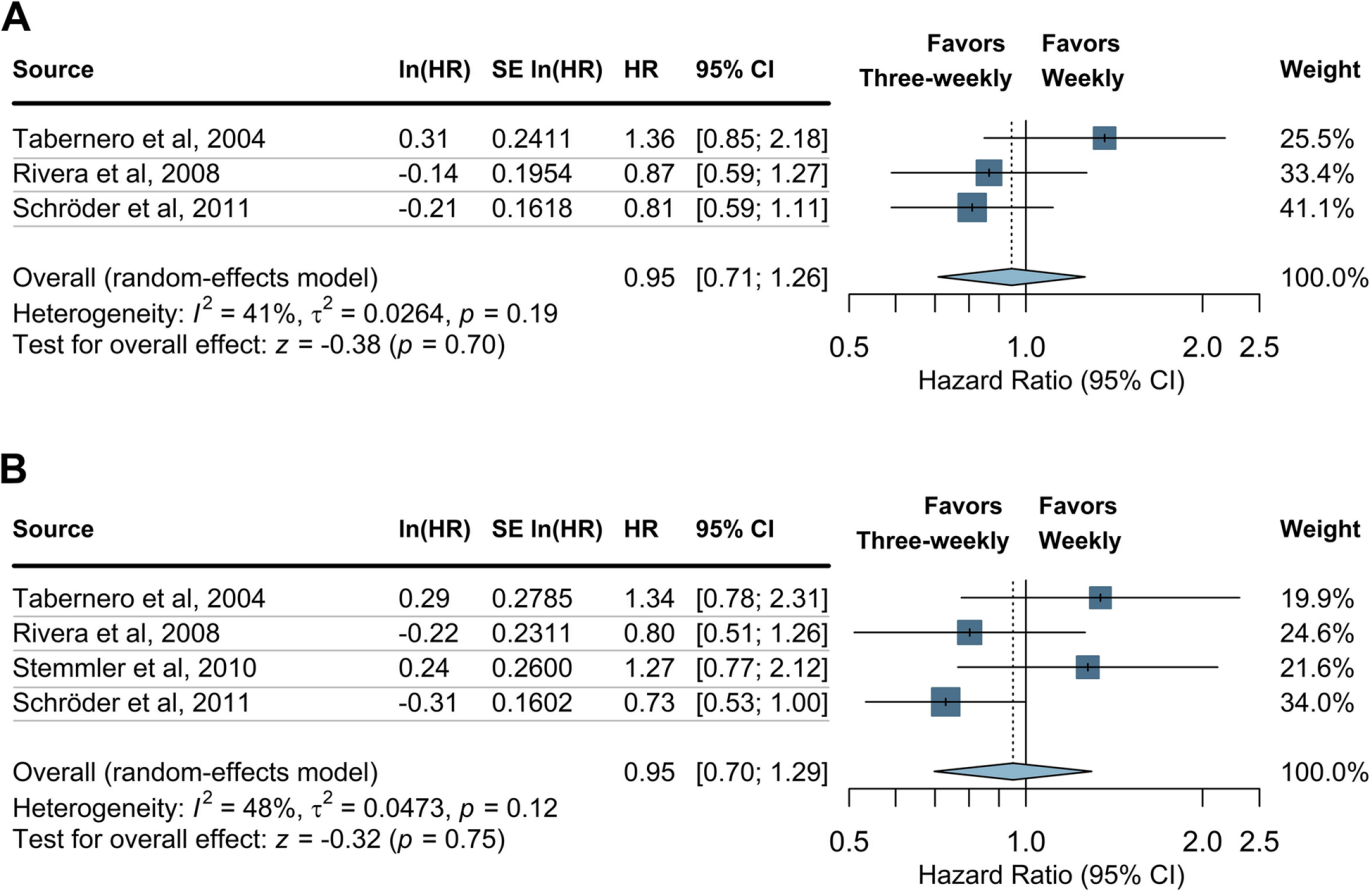
Random-effects meta-analysis of the hazard ratio of progression-free survival (A) and overall survival (B) comparing weekly and three-weekly docetaxel schedules; squares demonstrate the point estimate of the hazard ratio; lines represent the 95% CI; the size of each square is proportional to its weight in meta-analysis; diamonds represent the pooled estimate of the hazard ratio after meta-analysis; values < 1 indicate a lower chance of progression/death in the three-weekly arm as opposed to the weekly arm while values > 1 indicate a lower chance of progression/death in the weekly arm; I^2^ measure of statistical heterogeneity with the corresponding p-value a test for heterogeneity across studies; τ^2^ variance of the distribution of the observed study effects

The results of a random-effects meta-analysis of the RR of objective response rate according to an intention-to-treat analysis are shown in Fig. 2. In this analysis, no significant association between the weekly or three-weekly docetaxel schedule and objective response rate was seen (RR 0.75 95% CI: 0.54 – 1.05, *p* = 0.09) However, the direction of the overall effect indicates the possibility that the chance of an objective response might be lower in the weekly group than in the three-weekly group. For progression-free survival no significant association between weekly or three-weekly regimens was found (HR 0.95, 95% CI: 0.71—1.26, *p* = 0.70) (Fig. 3A). Similarly, no significant association between the weekly or three-weekly docetaxel schedule and overall survival was found (HR 1.05, 95% CI: 0.71—1.29, *p* = 0.75) (Fig. 3B).

### Risk of Bias

The risk of bias assessments for the included trials are included in the supplementary Figs. 1 and 2 (Additional file 2). All included RCTs had low risk of bias arising from the randomization process, bias due to missing outcome data, and bias in selection of the reported result. For one study, there was some concern for bias due to deviations from intended interventions since not all randomized patients were included in the final efficacy analyses [23]. In all studies, there was concern about risk of bias in the measurement of the outcomes. This was due to the timing of response and toxicity evaluations between the randomized groups. Patients were evaluated when visiting the hospital to receive the allocated treatment. The differences in applied schedules between the randomized groups within each trial inherently resulted in evaluations at different time points.

## Discussion

The results of our meta-analysis demonstrate that weekly docetaxel is associated with significantly less hematological and neurological toxicity while presenting with an increased risk of onycholysis and epiphora and a borderline significant increase in the risk of treatment withdrawal. Our analyses found no significant differences in objective response rate, progression-free survival or overall survival between both schedules.

A systematic review and meta-analysis with similar objectives has been performed by Mauri and colleagues in 2010 [26]. However, this meta-analysis included both paclitaxel and docetaxel studies and included only two of the RCTs included in the present analysis. With regard to toxicity, the results from our meta-analysis for docetaxel seem to be in line with the results that Mauri and colleagues found for both taxanes. We also find that, with respect to both hematological toxicity and efficacy, our outcomes are in accordance with the findings of previous meta-analyses which investigated weekly versus three-weekly docetaxel as second line treatment in non-small-cell lung cancer [27, 28]. In contrast to these previous studies, our meta-analysis demonstrated a difference in the risk of treatment withdrawal between weekly and three-weekly schedules. Although of borderline significance, this difference is important to note since it may affect the patient’s preference of docetaxel regimen and ultimately the outcome of docetaxel treatment.

We should note several limitations of our study. Firstly, despite our extensive literature searches, only four RCTs comparing weekly versus three-weekly docetaxel in mBC could be identified, comprising 459 patients. Secondly, as expected from the trial’s inclusion criteria, the study of Stemmler and colleagues had an older patient population as compared to the other 3 studies. Although patients with a poor performance status were allowed in this study, the Karnofsky Performance Status was still relatively high with a median of 80% in both arms [24]. Also, the prior treatments seemed less intensive and the administered docetaxel dose was lower as compared to the other three studies. In addition, Schröder and colleagues reported a substantially lower median overall survival for patients in both docetaxel arms compared to the other studies, which may indicate differences in the baseline patient populations between the four studies [25]. Moreover, baseline characteristics such as race, menopausal status and breast cancer subtype might affect tolerance and outcome of docetaxel treatment. Unfortunately, we do not have sufficient data on race, as this was provided in only 1 out of 4 studies [23]. Menopausal status was also not mentioned in the studies, except for the study of Stemmler et al., in which all patients were postmenopausal [24]. Therefore, although most patients are likely to have a prior chemotherapy-induced postmenopausal status, we do not have sufficient data on this. Although most studies provided the oestrogen receptor status, none of the studies described the percentage of patients with triple negative breast cancer.

As underlined by our risk of bias assessments, the frequency of toxicity assessments may have influenced individual trial results. In the study of Stemmler et al., blood counts were measured weekly [24]. While in the other 2 studies, all patients were evaluated for toxicity before every docetaxel infusion [22, 23]. This means that patients in the weekly arm were evaluated every 7 days, while with three-weekly docetaxel this occurred once every 21 days. For the trial of Schröder et al., the assessment frequency was not reported [25].

Since not all trials were uniform in their reporting of events, several assumptions were made to allow for pooled analysis of these outcomes. In some trials, nausea and vomiting was reported as a combined outcome while others reported these outcomes separately. We reasoned that, since patients who experience vomiting are likely to be nauseous, separate meta-analysis of these outcomes, with the event rate of the combined outcome counted in both separate analyses, would lead to the least biased result. In addition, for epiphora or lacrimation the event rates of ≥ grade 3 toxicity or toxicity leading to treatment withdrawal were included. While for onycholysis, the event rates of both ≥ grade 2 toxicities and toxicities leading to treatment withdrawal were included. We did not include the study of Stemmler and colleagues in the meta-analysis for neutropenia since the relevant hematological outcome reported in this study was leukopenia. However, the reported result for this similar outcome in their trial is in line with the result from our meta-analysis [24]. Furthermore, even though different weekly schedules were applied in the different included RCTs, we have decided not to perform any subgroup analyses because of the limited number of included trials. Lastly, as Mauri and colleagues have also stated, time to progression as reported by Tabernero and colleagues appears to be incorrect and is in fact progression-free survival [26]. Thus, in our analysis, the decision was made to analyze the reported time to progression as progression-free survival.

The four included RCTs are not unanimous regarding their preference for weekly or three-weekly docetaxel. The studies by Tabernero, Rivera, and Stemmler and colleagues conclude that, since efficacy in terms of survival benefit appears to be similar, the weekly schedule may be considered as an alternative to the conventional three-weekly schedule, specifically for patients at higher risk for hematological toxicity [22–24]. In contrast, Schröder et al. clearly prefer the three-weekly regimen, since the non-hematological toxicities (fatigue and general malaise) with weekly docetaxel led to more treatment withdrawals, despite a lower frequency of neutropenia. The lower treatment completion in this trial even led to a lower overall survival with weekly docetaxel in multivariate analysis [25].

The marked difference in risk of hematological toxicity between weekly and three-weekly docetaxel schedules may be attributable to pharmacokinetic, pharmacodynamic, or schedule-related differences. The clearance of docetaxel has shown to be the strongest predictor for the development of severe neutropenia, after evaluation in 640 patients from 24 phase II studies treated with first course 75–100 mg/m^2^ docetaxel [29]. Not surprisingly given the linear pharmacokinetics, the clearance with weekly (35 mg/m^2^) and three-weekly (60–100 mg/m^2^) docetaxel in 46 patients with advanced solid tumors, was similar [30]. The total cumulative exposure, as calculated as the area under the plasma concentration versus time curve (AUC) over a three-week period, was also comparable with weekly and three-weekly docetaxel [30]. In addition, the maximum plasma concentration (C_max_) with weekly docetaxel (35 mg/m^2^, infused in 30 min) was comparable to the three-weekly dose of 75 mg/m^2^ (infused in 1 h) [30]. Therefore, the lower neutropenia rates with weekly as compared to three-weekly schedules are unlikely related to large differences in docetaxel clearance, cumulative AUC per cycle or C_max_.

Pharmacodynamic factors known to increase the sensitivity for docetaxel-related neutropenia, are a low baseline neutrophil count and a higher number of previous chemotherapy regimens [29]. Moreover, this sensitivity seems different between patient groups. At direct comparison of patients aged ≤ 65 years versus > 65 years, the clearance of docetaxel (dosed 75 mg/m^2^) was unaltered, but the neutropenia rate was much higher in the older group (grade 4 neutropenia 63% (*n* = 19) vs 30% (*n* = 20) and febrile neutropenia 16% vs 0%, respectively) [31]. Also with the weekly schedule, non-hematological toxicities seem more prevalent in patients aged ≥ 65 years, as 10 out of 19 evaluable patients experienced ≥ grade 3 non-hematological toxicities in a study with weekly docetaxel (35 mg/m^2^), without any age-related differences in the pharmacokinetics [32]. Lastly, despite similar pharmacokinetics, the neutropenia rates are higher in Japanese patients treated with docetaxel 60 mg/m^2^ as compared to Western patients treated with 75–100 mg/m^2^ doses. The reason for this increased sensitivity to docetaxel-induced neutropenia remains to be elucidated [33]. Nonetheless, the difference in the rate of hematological toxicity between treatment arms was present in all trials included in our meta-analysis despite their randomized nature and well balanced populations per treatment arm [22–25]. Which therefore makes it unlikely an effect of individual patient characteristics.

With respect to schedule, weekly docetaxel allows for more opportunities for dose delays, reductions, or omissions within a cycle when patients experience treatment related toxicities such as neutropenia compared to three-weekly administration. Moreover, the more frequent blood counts and hospital visits that are inherent to a weekly schedule may lead to an earlier detection of toxicity and lead to the implementation of such dose adjustments, thereby potentially preventing worse grade toxicities. This seems to be in agreement with the fact that some trials reported a decreased dose-intensity [23, 25] or an increased rate of omitted doses in the weekly arm [24].

However, inherent to the weekly docetaxel schedule are also a higher burden of more frequent hospital visits and intravenous administrations for patients. From that perspective, the development of oral docetaxel is an attractive alternative [34].

## Conclusions

In this meta-analysis of patients with mBC the safety and efficacy of weekly versus three-weekly docetaxel was investigated. Overall, the risk of grade 3/4 neutropenia, febrile neutropenia and neuropathy was significantly lower with weekly docetaxel while no significant differences in efficacy were found compared to the three-weekly schedule. However, the risk of onycholysis, epiphora and toxicity leading to treatment withdrawal seems to be increased with weekly docetaxel. For specific patient populations vulnerable to neutropenia, such as older patients, patients with a high number of prior chemotherapy treatments or low baseline neutrophil levels, weekly docetaxel might be favorable. However, three-weekly docetaxel offers the same efficacy with a lower burden of hospital visits for patients. Therefore, both schedules may be considered and applied based on the preference of the individual patient.

## Supplementary Information

**Additional file 1.** The full Medline (Ovid), Embase (Ovid) and Scopus search strategies used for in the literature searches.

**Additional file 2.** Visualization of the outcomes of risk of bias assessments of included randomized controlled trials.

## Data Availability

The datasets used and/or analyzed during the current study can be made available from the corresponding author upon request.

## Abbreviations

AUC: Area under the plasma concentration versus time curve
CI: Confidence interval
C_max_: Maximum plasma concentration
HR: Hazard ratio
lnHR: Log transformed hazard ratio
mBC: Metastatic breast cancer
NR: Not reported
PRISMA: Preferred Reporting Items for Systematic Reviews and Meta-Analyses
PS: ECOG Performance status
QW: Once weekly
Q3W: Three-weekly
RCT: Randomized controlled trial
RR: Risk ratio
SE: Standard error
