# Supplementary material for: The influence of docetaxel schedule on treatment tolerability and efficacy in patients with metastatic breast cancer: a systematic review and meta-analysis of randomized controlled trials
Source: BMC Cancer. 2022 Jan 25;22:104. doi: 10.1186/s12885-022-09196-x (PMC8788086; doi:10.1186/s12885-022-09196-x)
Supplement: Supplementary file 1 — Additional file 1. The full Medline (Ovid), Embase (Ovid) and Scopus search strategies used for in the literature searches. [file 12885_2022_9196_MOESM1_ESM.pdf]

Supplementary material to: “*The influence of docetaxel schedule on treatment tolerability and efficacy in patients with metastatic breast cancer: a systematic review and meta-analysis of randomized controlled trials.*”
M. van Eijk, M. Vermunt, E. van Werkhoven, E.A. Wilthagen, A.D.R. Huitema, J.H. Beijnen
BMC Cancer
M. van Eijk
Department of Pharmacy & Pharmacology,
Antoni van Leeuwenhoek – The Netherlands Cancer Institute.
maa.v.eijk@nki.nl

Description: The full Medline (Ovid), Embase (Ovid) and Scopus search strategies used for in the literature searches

Additional file 1. Medline (Ovid), Embase (Ovid) and Scopus search strategies

Medline (Ovid)

("Survival Analysis"[Mesh] OR "Survival Rate"[Mesh] OR (Survival[tiab] AND (rate[tiab] OR rates[tiab] OR "disease free"[tiab] OR overall[tiab] OR "event free"[tiab] OR "progression free"[tiab] OR analys*[tiab] OR analyz*[tiab])) OR "objective response rat*" [tiab] OR "overall response rat*" [tiab] OR ORR [tiab] OR "time to progres*" [tiab] OR OS[tiab] OR PFS[tiab] OR EFS[tiab] OR DFS[tiab] OR TTP[tiab]) AND ("Breast Neoplasms"[Mesh] OR ((breast*[tiab] OR mammary[tiab]) AND (neoplasm* [tiab] OR tumor [tiab] OR tumors[tiab] OR tumour*[tiab] OR cancer*[tiab] OR malign*[tiab] OR oncolog*[tiab] OR carcinom*[tiab] OR lymphom*[tiab])) OR mamacarcinom* [tiab] OR mammacarcinom* [tiab] OR mamma-carcinom* [tiab] OR MBC [tiab]) AND ("docetaxel"[Supplementary Concept] OR docetaxel[tiab] OR Taxotere[tiab] OR "NSC 628503"[tiab] OR "RP 56976"[tiab] OR N-debenzoyl-N-tert-butoxycarbonyl-10-deacetyltaxol[tiab] OR DTX[tiab])

Embase (Ovid)

(exp survival analysis/ or exp survival rate/ or ((Survival adj3 (rate or rates or disease free or overall or "event free" or "progression free" or analys* or analyz*)) or "objective response rat*" or "overall response rat*" or ORR or "time to progres*" or OS or PFS or EFS or DFS or TTP).ti,ab,kw.) and (exp breast cancer/ or (((breast* or mammary) adj3 (neoplasm* or tumor or tumors or tumour* or cancer* or malign* or oncolog* or carcinom* or lymphom*)) or mamacarcinom* or mammacarcinom* or mamma-carcinom* or MBC).ti,ab,kw.)

and (exp docetaxel/ or (docetaxel or Taxotere or "NSC 628503" or "RP 56976" or N-debenzoyl-N-tert-butoxycarbonyl-10-deacetyltaxol or DTX).ti,ab,kw.)

Scopus

(TITLE-ABS-KEY(docetaxel OR taxotere OR "NSC 628503" OR "RP 56976" OR n-debenzoyl-n-tert-butoxycarbonyl-10-deacetyltaxol OR dtx)) AND (TITLE-ABS-KEY(((breast* OR mammary) W/2 (neoplasm* OR tumor OR tumors OR tumour* OR cancer* OR malign* OR oncolog* OR carcinom* OR lymphom*)) OR mamacarcinom* OR mammacarcinom* OR "mamma-carcinom*" OR mbc)) AND (TITLE-ABS-KEY((survival W/2 (rate OR rates OR "disease free" OR overall OR "event free" OR "progression free" OR analys* OR analyz*)) OR "objective response rat*" OR "overall response rat*" OR orr OR "time to progres*" OR os OR pfs OR efs OR dfs OR ttp))
