# Supplementary material for: The influence of docetaxel schedule on treatment tolerability and efficacy in patients with metastatic breast cancer: a systematic review and meta-analysis of randomized controlled trials
Source: BMC Cancer. 2022 Jan 25;22:104. doi: 10.1186/s12885-022-09196-x (PMC8788086; doi:10.1186/s12885-022-09196-x)

Additional file 2
Supplementary material to: "*The influence of docetaxel schedule on treatment tolerability and efficacy in patients with metastatic breast cancer: a systematic review and meta-analysis of randomized controlled trials.*"
M. van Eijk, M. Vermunt, E. van Werkhoven, E.A. Wilthagen, A.D.R. Huitema, J.H. Beijnen
BMC Cancer
Corresponding author:
M. van Eijk
Department of Pharmacy & Pharmacology,
Antoni van Leeuwenhoek – The Netherlands Cancer Institute.
maa.v.eijk@nki.nl

Description: Visualization of the outcomes of risk of bias assessments of included randomized controlled trials.

Supplementary Figure 1. Summative plot of assessment of risk of bias using the Cochrane RoB2 tool

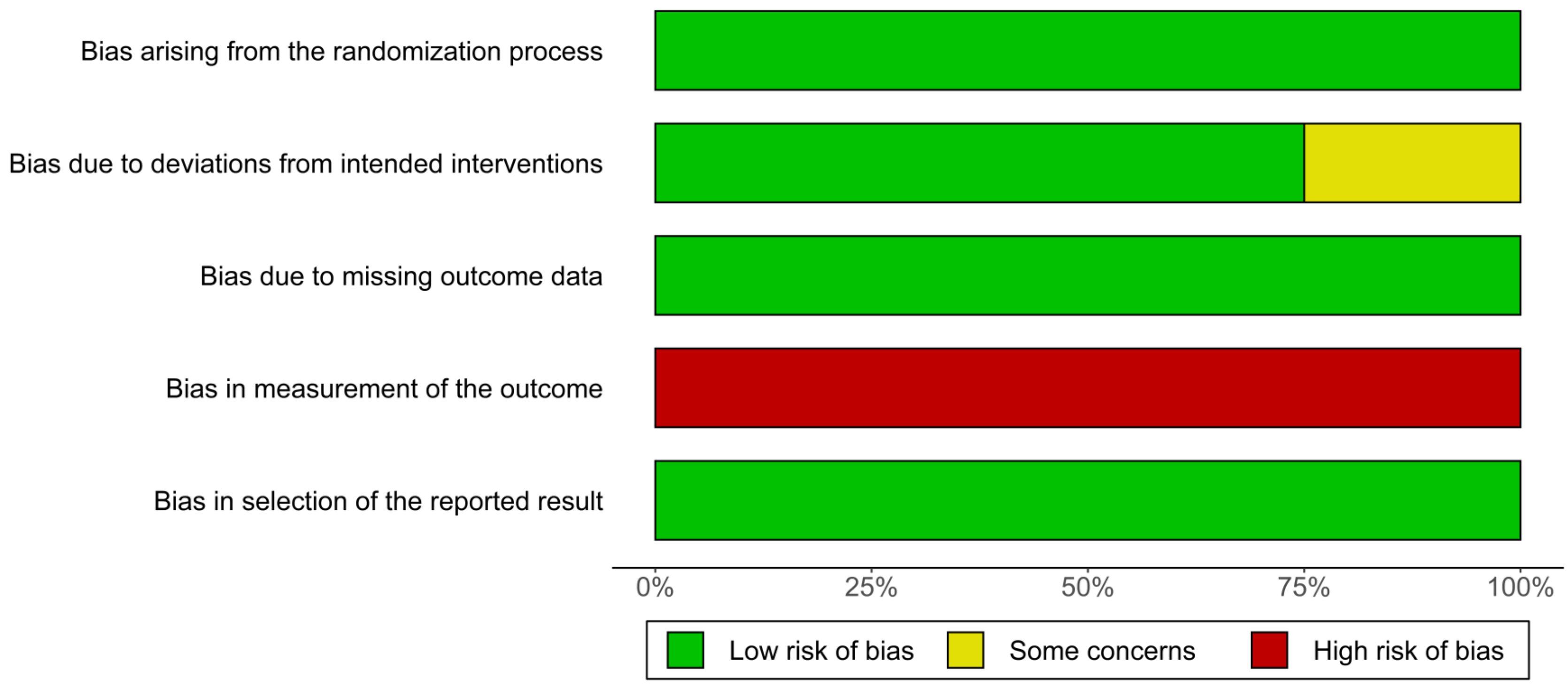

Supplementary Figure 2. Traffic light plot of assessment of risk of bias using the Cochrane RoB2 tool

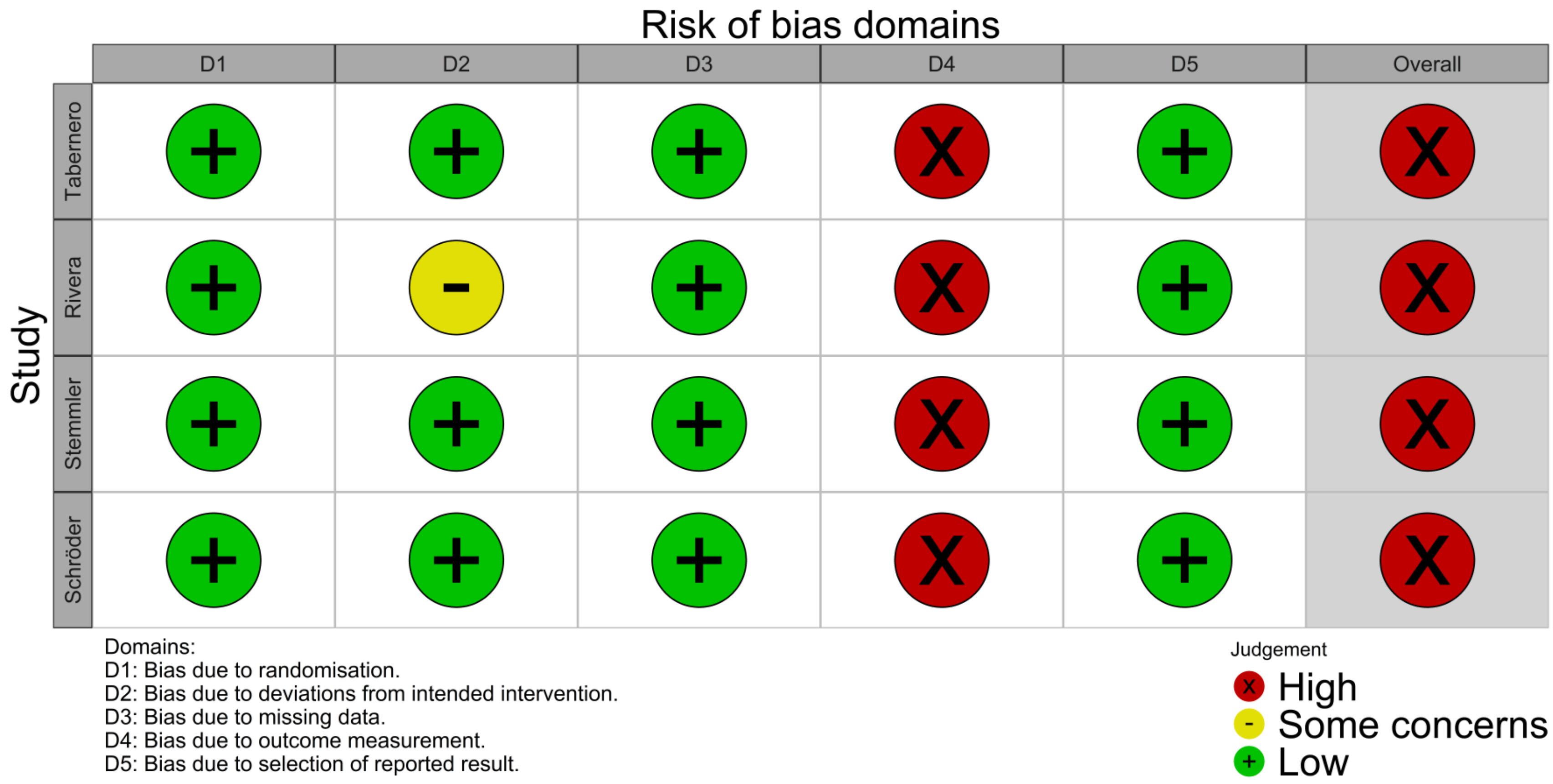

Supplement: Supplementary file 2 — Additional file 2. Visualization of the outcomes of risk of bias assessments of included randomized controlled trials. [file 12885_2022_9196_MOESM2_ESM.pdf]
